# Molybdenum targets produced by mechanical reshaping

**DOI:** 10.1007/s10967-015-3956-1

**Published:** 2015-02-15

**Authors:** A. Stolarz, J. A. Kowalska, P. Jasiński, T. Janiak, J. Samorajczyk

**Affiliations:** 1 Heavy Ion Laboratory, University of Warsaw, ul. Pasteura 5a, 02-093 Warsaw, Poland; 2 Radioisotope Centre POLATOM, National Centre for Nuclear Research, ul. Andrzeja Sołtana 7, 05-400 Otwock, Poland; 3 Faculty of Physics and Applied Informatics, ul. Pomorska nr 149/153, 90-236 Łódź, Poland

**Keywords:** Molybdenum, Rolling, Technetium

## Abstract

Targets required to determine the parameters of the ^100^Mo(p,xn)^99m^Tc reaction and to estimate the yield of the ^99m^Tc production were prepared starting with powder material. Material, melted with electron beam gun into solid bead, was reshaped into foil mechanically. Targets were prepared by powder melting and hot flattening of the droplet followed by cold rolling. Procedure allowed preparation of thick (in the range of hundreds of microns) and thin (down to 250 nm) foils.

## Introduction

The metastable ^99m^Tc widely applied as radioactive tracer in medical diagnostic procedures currently is mainly obtained from the molybdenum-99 (^99^Mo) in its radioactive decay. The ^99^Mo is produced by irradiation of enriched ^235^U with flux of neutrons provided by research reactors.

$$^{235}{\text{U}} + {\text{n}} \,{\rightarrow}\,^{99}{\text{Mo}}\, {\mathop{\rightarrow}\limits^{\upbeta}}\,^{99\text{m}} {\text{Tc}} \,{\mathop{\rightarrow}\limits^{\upgamma}}\,^{99}{\text{Tc}}$$

The ^99^Mo produced in the above reaction is extracted from target and after purification is delivered to hospitals where is used as generator of ^99m^Tc.

The reactors used to supply the ^99^Mo were build 40–50 years ago and recently world customers had to face not only planned but as well unexpected shut downs of some of reactors what caused shortages in the ^99^Mo and thus ^99m^Tc supply.

^99^Mo, source of ^99m^Tc, can be produced also by neutron capture in ^98^Mo inserted into the core of a nuclear reactor so this method, although considered as alternative for use of HEU, requires reactors as well what is a significant drawback when assessing usefulness of ^99m^Tc production this way. Other drawbacks are discussed in [1].

Thus, the growing problem with operationality of research reactors (interruptions of their work) stimulated search for alternative ways of ^99m^Tc production either via production of ^99^Mo [2] or direct production of ^99m^Tc [3, 4] although the last solution due to the isotope half-life can be seen as alternative for local supplies only [5]. Both isotopes can be produced in accelerators providing protons, deuterons, alpha projectiles using various Mo isotopes as targets (Table 1), but direct ^99m^Tc production in reaction of ^100^Mo with protons is considered as the most promising (due to its cross section, production energy range) alternative way of ^99m^Tc production. Advantages and drawbacks of this solution are presented in many publications [e.g. 3, 5] and they will not be discussed in this paper as it is aside of the work objective.

**Table 1:** Possible accelerator based reactions to produce ^99m^Tc or ^99^Mo

^99m^Tc	^99^Mo
^100^Mo(p,2n)^99m^Tc	^100^Mo(p,x)^99^Mo
^100^Mo(d,3n)^99m^Tc	^100^Mo(d,x)^99^Mo
^98^Mo(p,γ)^99m^Tc	^100^Mo(γ,n)^99^Mo
^98^Mo(d,n)^99m^Tc	^100^Mo(p,2p)^99^Nb → ^99^Mo
^97^Mo(d,γ)^99m^Tc	^98^Mo(d,p)^99^Mo
^96^Mo(α,p)^99m^Tc	^97^Mo(α,2p)^99^Mo
	^96^Zr(α,n)^99^Mo

**Fig. 1:**
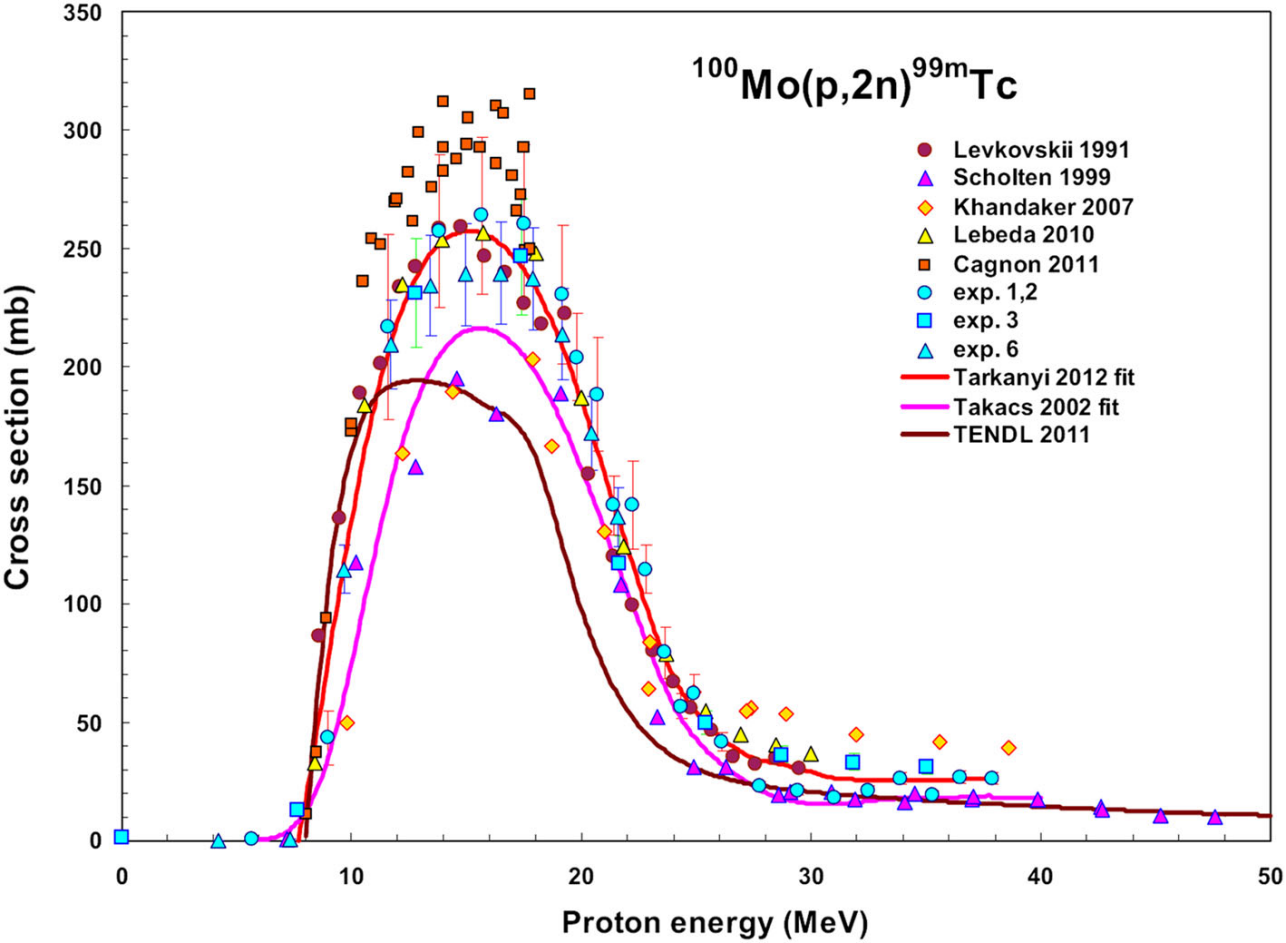
Excitation function of the ^100^Mo(p,2n)^99m^Tc reaction [10]

The excitation function of ^100^Mo(p,2n)^99m^Tc reaction has been studied by many authors for decades ([6–9] just to list few) but nevertheless the value of cross section of this reaction is still not well defined. The measured values of the excitation function of the proton-induced reactions on molybdenum obtained by different researchers are presented in Fig. 1. As can be seen from the plot, values presented by different authors differ even by factor of 2. It is difficult to point out all sources of this inconsistence but it is not excluded that one of them is related to the fact that in the most cases the cross section studies were completed with natural material. The excitation function expected for isotopically enriched Mo was then estimated based on the results obtained for ^nat^Mo (^92^Mo—14.84 %, ^94^Mo—9.25 %, ^95^Mo—15.92 %, ^96^Mo—16.68 %, ^97^Mo—9.55 %, ^98^Mo—24.13 %, ^100^Mo—9.63 %) [e.g. 8].

This scatter of the cross section values motivated us to study the cross section of the ^100^Mo(p,2n)^99m^Tc reaction by direct measurement of the excitation function using targets of the enriched ^100^Mo to avoid errors resulting from estimation of the cross section for ^100^Mo based on results obtained with natural Mo.

## Procedure of target preparation

Isotopically enriched molybdenum is available in powder form and thus studying the excitation function of the discussed reaction required conversion of this material into a foil of relatively low thickness, while studies of the reaction yield require thick targets.

There are many methods applied to prepare thick targets of molybdenum on backings for ^99m^Tc production for medical use:

- Electrophoretic deposition plus high temp (1 600 °C) sintering in H_2_ atmosphere (described in [11]),
- Powder pressing as self standing pellet followed by sintering, brazing or pressing into backing, or powder pressing into backing [12, 13],
- ‘Foil’ forming by direct powder rolling [14],
- Thermal cladding–laser plating [15],
- Forming low melting Mo alloys [16].

However, ^99m^Tc production with target in solid metallic form could be favourable considering its better thermal conductivity comparing to powdered targets, what may allow the use of higher beam intensity.

Taking into account the form of the available enriched ^100^Mo, our procedure of preparation of the metallic foils consists of powder consolidation by melting and then the bead conversion into a foil by mechanical reshaping.

### Powder consolidation by melting

**Fig. 2:**
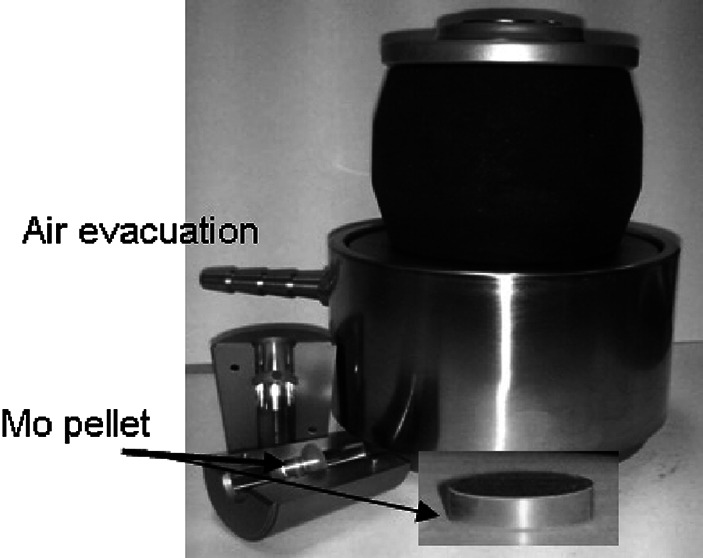
The pellet die with air evacuation option

The powdered material in the amount corresponding to the target thickness and its size (up to about 1 300 mg) was pelletized with use of a die allowing the air removal during pellet forming (Fig. 2), and a hydraulic press.

**Fig. 3:**
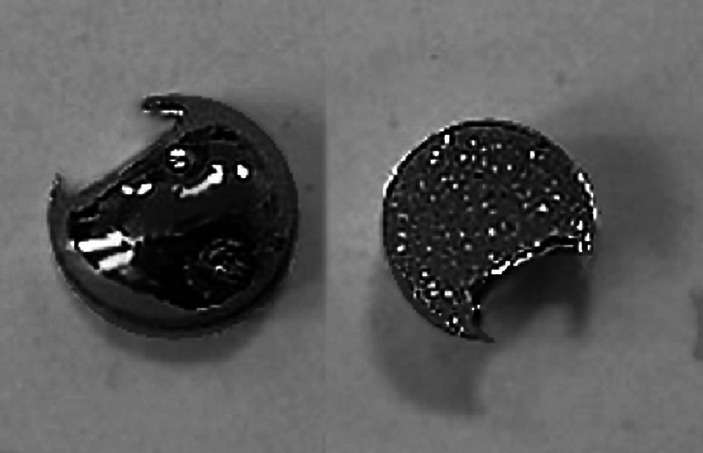
Partially melted molybdenum pellet made of 1 350 mg of the Mo powder

The obtained pellet was melted into a droplet in the vacuum of ~10^−6^ mbar with e-beam gun. Before reaching the melting temperature, pellet was carefully heated with e-beam, both for outgassing, i.e. removing the air residual, and evaporating the molybdenum oxide (*t*_evap_ = ~1 155 °C). The e-beam intensity was increased gradually until stable pressure of ~10^−6^ mbar was reached. Only then the e-beam intensity was increased to melt the Mo pellet into a droplet. In case of thicker pellets only the upper part was melted in the first run and formation of droplet was completed after breaking the vacuum and turning the half melted pellet upside-down (Fig. 3).

Further re-melting of the received droplet is required to prepare a bead of good for rolling quality (smooth, without deformation that can act as a starting point of droplet cracking when rolled). Re-melting of the material has to be done with changing of its position in the crucible of e-beam gun, i.e. turning the bead to expose each side to the electron beam. It is important especially in case of droplets made of big amount of material (few hundreds milligram). The total material loss during melting process was of about 15–18 %.

### Material reshaping

#### Rolling

**Fig. 4:**
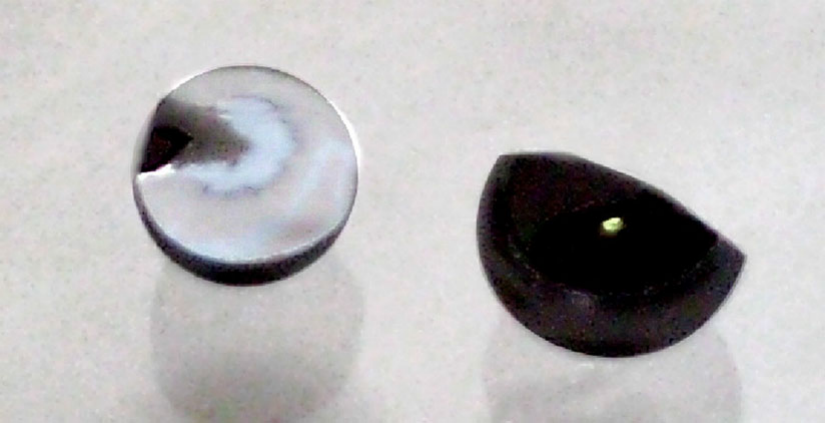
Molybdenum bead after first pass through the rolling mill set too tightly

Droplet produced by powder melting was placed between stainless steel sheets (rolling pack) and passed through the rolling mill. The applied rolling speed was of about 10 RPM (125 cm min^−1^) and thickness reduction was not greater than 4–5 µm at the initial steps irrespective the size of the droplet/disc. Higher reduction of the thickness would result with inevitable droplet crack at first pass through the rolling mill (as reported by [17] and others), see Fig. 4.

Below 0.5 mm the thickness reduction was not bigger than ~2.5 µm, otherwise the rolled material emerged as disc/foil with many cracks or as small, inutile pieces, too small to produce even the thin (10 μm) foils. During rolling process material, after each change of the rollers distance, was passed 4–5 times through rolling mill.

**Fig. 5:**
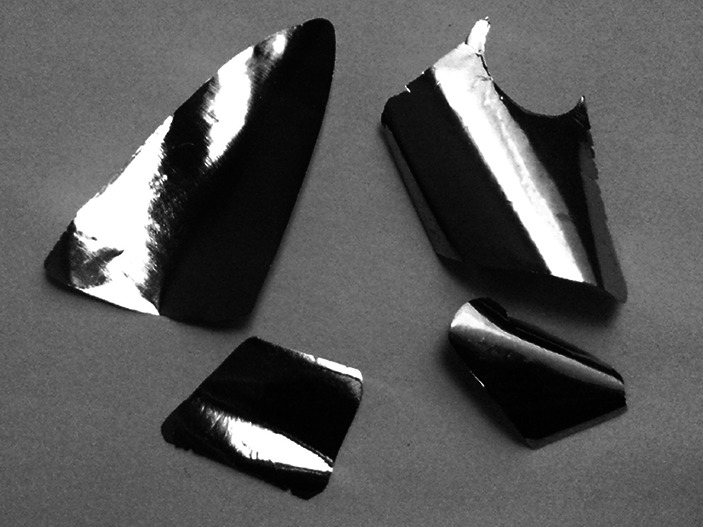
The 10 μm thick ^nat^Mo foils prepared with (*left*) and without (*right*) annealing. Not only the foil bending but as well the small cracks on the edge of not annealed foil can be observed

To remove stresses from the rolled foils they were annealed in vacuum for ~10–15 min at temperature of ~1 200 °C. The influence of the annealing on the foils properties can be seen in Fig. 5.

Described procedure allows production of thin (10 µm) foils. The production of sufficient area of these foils (to prepare stacked foil target composed of 10 Mo pieces) took about 1 week of the whole day work.

**Fig. 6:**
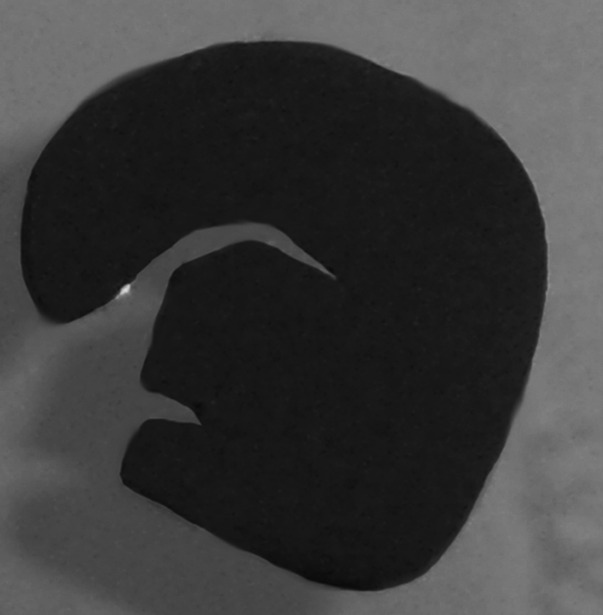
Example of crack passing through the disc of ~1 mm thick

Annealing useful at preparation of thin foils (below 100 µm) was not significantly helpful in production of thick ones (400–600 µm). The amount of cracks was lower but, when appearing, they propagated through the foil area preventing production of the foil of the required size (Fig. 6).

Lipski [18] suggests that slow reduction of the e-beam intensity should reduce the stresses in the material and decrease crackability but such relation was not observed in case when big droplets needed for thick target preparation were produced. Not only droplets but also thick discs and plates were cracking with the same ‘easiness’ irrespective of slow cooling of the melted material in case of small droplets. Slow cooling of big droplets, as mainly described in this work, resulted with brittle material.

N. Y. Kheswa in her paper [19] reports production of malleable, not cracking molybdenum droplet just by thorough melting but the amount of material used in [19] (only 75 mg of the starting amount) is incomparably smaller than the amount required by our needs (one target of 1.4 cm × 1.4 cm of 600 µm thick requires ~1 300–1 400 mg of molybdenum). Thorough melting, at a single run, of the amount of Mo as used by Kheswa seems to be easier. The cold flattening recommended by K. Zell [20], applied by him to the droplet of ~2 mm in diameter most probably does not stress the material at the same level as in case of droplet of 6–7 mm in diameter made of 1 300–1 400 mg of starting amount of Mo.

Substantial material loss (40 %) reported in [19] is not acceptable as well in case of thick targets of expensive material such as ^100^Mo. There is also no information on thickness and size of the produced foils so the final result can not be compared to our work.

**Fig. 7:**
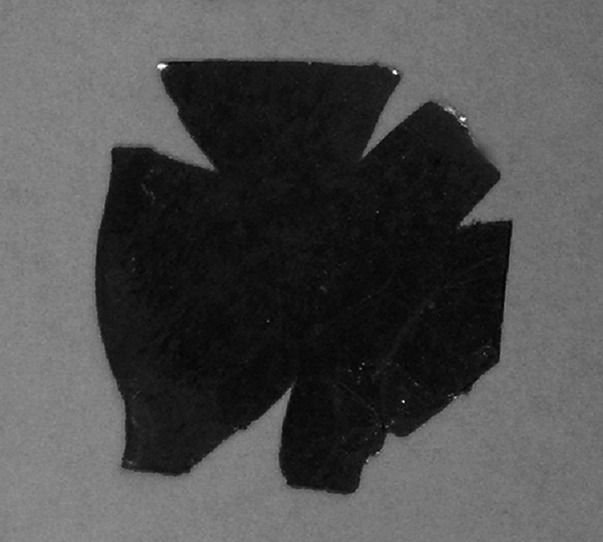
The 80 μm thick foil produced from the droplet obtained by melting the Mo pellet beforehand sintered at 1 600 °C under the hydrogen atmosphere

Expecting improvement of the purity of the melted material, and thus its malleability, the Mo powder was heated in the reducing atmosphere (1 h at 1 600 °C at H_2_ atmosphere) for removing the oxide residues before pellet forming. At other approach the pellet was sintered under mentioned condition but no improvement of the molybdenum malleability was observed. On the contrary, the droplet resulting from the pre-treated powder was less malleable. The Fig. 7 shows the foil prepared using the droplet produced from the powder sintered in the above listed condition.

#### Hot reshaping of the droplet and subsequent cold rolling

To produce thick foils, the relatively big droplets (6–7 mm diameter) were flattened in high temperature before rolling.

**Fig. 8:**
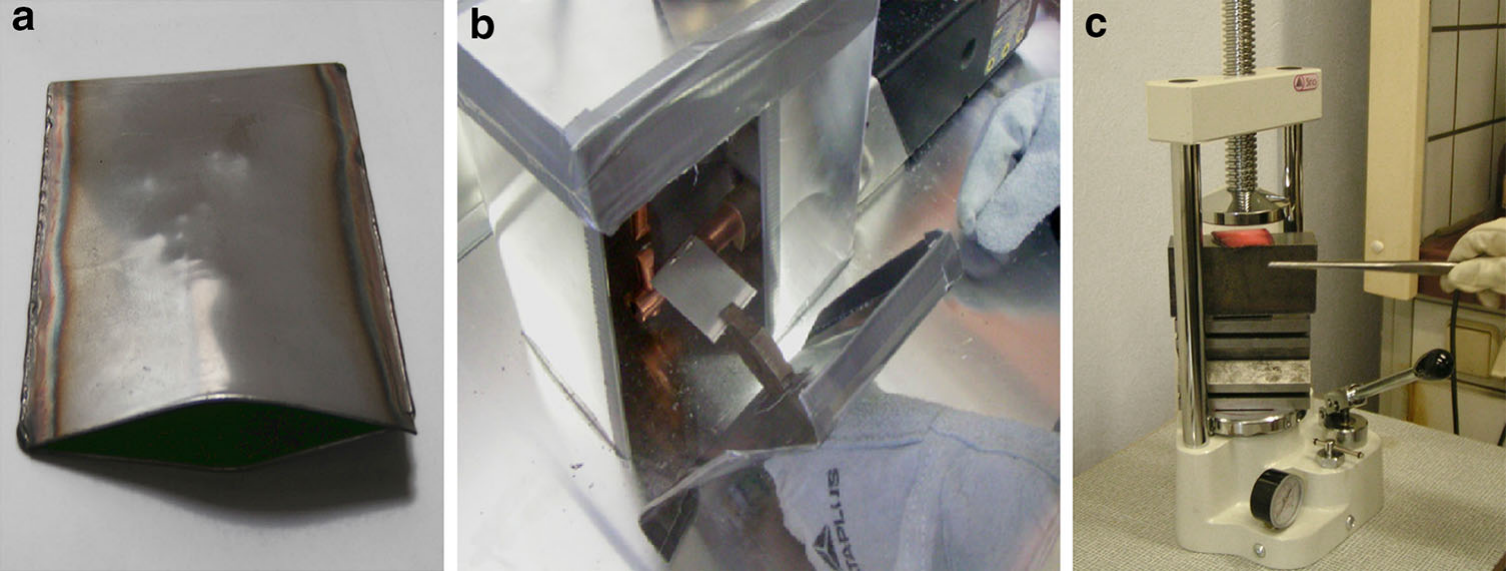
To flatten the Mo droplet in the high temperature, droplet prepared by powder melting was packed into the stainless steel envelope (**a**) and sintered tightly under the argon atmosphere (**b**). The packet was heated up to 1 100 °C and when hot, pressed under the hydraulic press (**c**)

Molybdenum, oxygen resistant metal at ambient temperature, oxidises easily at temperature above 600 °C. To protect molybdenum from oxidation at elevated temperature, the Mo droplet was packed into the stainless steel packet (envelope) under argon atmosphere (Fig. 8a, b).

The packed droplets of ~6–7 mm in diameter were heated at temperature of 1 100 °C for 3–5 min and when hot were flattened with the use of hydraulic press as quickly as possible to preserve the high temperature (see Fig. 8c). The height of the droplet and further of the disc was reduced by 20–25 % at initial steps and by ~15 % in consecutive steps until disc was about 1–1.5 mm thick. Example of the forces used for flattening is given in the Table 2.

**Table 2:** Examples of droplets reshaped by hot flattening

Sample no; initial amount (% material loss at melting)	Pressure (bar)	Comments
Sample 6/1 ^nat^Mo 1 041.46 mg (~20 %)	1st 60 2nd 80 3rd 130	Size after hot press: 7 × 7.5 × 2.5 mm, slightly oxidised, after oxide removal by e-beam heating, additional cold press was applied: 8.65 × 8.35 × 1.8–1.4 mm (small crack on the rim) Part of this sample was rolled down to 250 nm with thickness reduction by 2.5–3 µm
Sample 8/3 ^nat^Mo 708.73 mg (~15 %)	1st 140 2nd 180 3rd 180	No visible oxidation; only hot press, appears to be easier to roll than sample 6/1 but final thickness similar to sample 6/1, ~280 nm
Sample E4/1 ^100^Mo 1 387.182 mg (~13 %)	1st 120 2nd 160 3rd 180 4th 180	Only hot press, thick.: 1.5–1.65 mm, size: 0.95 × 1 cm, very malleable, rolled only down to required target thickness i.e. 600 µm, the foil of 600 µm (1.5 × 2.5 cm) obtained with only one crack on the edge
Sample E5/2 ^100^Mo 1 337.494 mg (~15 %)	1st 140 2nd + 3rd 180 4th 200 5th 220	Only hot press; thickness: 1.5–1.3 mm, size: 1 × 1.2 cm, cracking easier than E4/1, rolled down to 410 µm (was there too high force at flattening?)

**Fig. 9:**
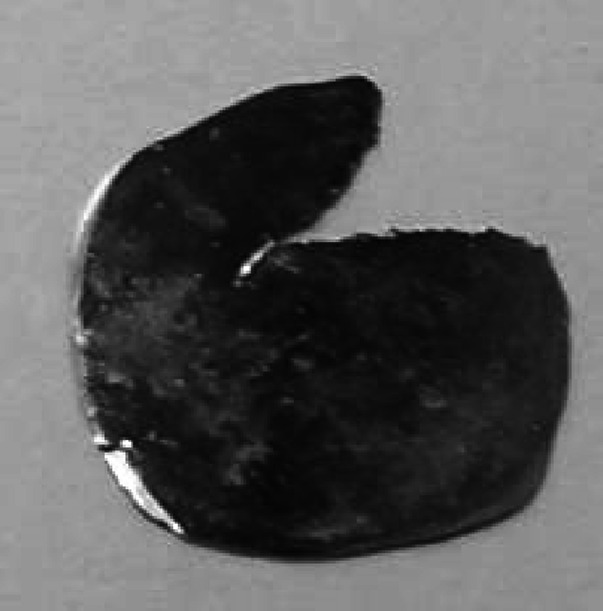
^nat^Mo after hot flattening and cold rolling down to 320 μm

After last flattening, the packet was left under argon atmosphere for cooling down. When cold, disc was removed from the envelope and rolled down to the required thickness of few hundreds micrometer. The Fig. 9 shows the foil of 320 µm with crack free area (~1.5 cm × 1.6 cm) sufficient for the target. But as can be seen in Table 2 (sample E4/1), foils of 600 µm of bigger area (1.5 cm × 2.5 cm) with only single crack, 2–3 mm long, were prepared from later produced droplets of ^100^Mo.

The foil was prepared from droplet of 7 mm diameter. The upper part of the presented foil was used to produce thinner, 10 µm thick foils needed to build stacked foil target.

## Conclusions

**Fig. 10:**
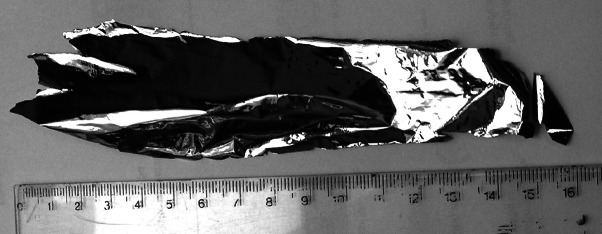
The 4.5–5.5 µm foil of 3 cm × 15 cm produced from natural Mo

Big molybdenum beads (6–7 mm in diameter made of more than 1 g of the material), prepared for rolling by powder melting with e-beam gun and hot flattening of the received droplet, demonstrated better malleability than only thoroughly melted material. It was possible to produce the thin foils in much shorter time than in the case of material prepared by melting only. The thickness reduction per pass was of similar value but number of passes per reduced thickness required to get ‘no size changeable’ foil significantly dropped down. Described procedure allows not only production of thick foils free from cracks but makes also possible to produce the thin foils of big area (Fig. 10).

The thinnest foil produced at this work was of ~250 nm (thickness measured by alpha particle energy loss method [21]). Below this thickness the material starts sticking to the rolling pack and tries to further reduce the foil thickness were not undertaken. The main aim of this work was to develop the procedure of production of thick (few hundreds micrometer) and thin (10 µm) Mo foils/plates of area of ~1.5 × 1.5 cm, thus the possibilities of further thinning of the foil were not investigated. It is not excluded that an application of anti-adhesive agent such as e.g. Teflon as rolling pack lining would allow reduction of the foil thickness.

The hot reshaping of the Mo droplet in the way described above, applied before cold rolling, is relatively simple. The Mo material after cooling down can be easily removed from the envelope and sticking to the stainless steel as reported by Karasek [17] at hot rolling applied by him was not observed.
